# Reduced association of anti-apoptotic protein Mcl-1 with E3 ligase Mule increases the stability of Mcl-1 in breast cancer cells

**DOI:** 10.1038/bjc.2011.242

**Published:** 2011-07-05

**Authors:** S Pervin, A Tran, L Tran, R Urman, M Braga, G Chaudhuri, R Singh

**Affiliations:** 1 Department of Internal Medicine, Charles Drew University of Medicine and Science, 3084 Hawkins Building, 1731 East 120th Street, Los Angeles, CA 90059, USA; 2 Department of Obstetrics and Gynecology, David Geffen School of Medicine at UCLA, 22-115 CHS, 10833 Le Conte Avenue, Los Angeles, CA 90095-7358, USA; 3 Jonsson Comprehensive Cancer Center, David Geffen School of Medicine at UCLA, 10833 Le Conte Avenue, Los Angeles, CA 90095-7358, USA

**Keywords:** apoptosis, ubiquitin, proteosome, E3 ligase

## Abstract

**Background:**

Mechanisms that increase resistance to apoptosis help promote cellular transformation. Cancer cells have deregulated apoptotic pathways, where increased expression and stability of anti-apoptotic proteins Mcl-1 and Bcl-2 increases resistance to apoptosis. Pathways that increase the stability of proteins in cancer cells remain poorly understood.

**Methods:**

Using human mammary epithelial and established breast cancer cell lines, we assessed the mechanisms that increase the stability of anti-apoptotic proteins in breast cancer cells by caspase assay, western blot, small-inhibitory RNA treatment and immunoprecipitation.

**Results:**

While breast cancer cells were resistant to *de novo* inhibition of protein synthesis, a rapid proteosome-mediated degradation of Mcl-1 and Bcl-2 induced apoptosis in mammary epithelial cells. Although Mule, an E3 ligase that targets Mcl-1 for degradation was expressed in mammary epithelial and breast cancer cell lines, rapid increase of polyubiquitinated Mcl-1 and Bcl-2 was detected only in mammary epithelial cells. Only transient formation of the Mule–Mcl-1 complex was detected in breast cancer cells. Downregulation of pERK1/2 in breast cancer cells reduced Mcl-1 levels and increased Mcl-1/Mule complex.

**Conclusion:**

Our findings suggest that reduced Mule/Mcl-1 complex has a significant role in increasing the stability of Mcl-1 in breast cancer cells and increased resistance to apoptosis.

Progression of tumourigenesis is dependent upon many factors including mechanisms that increase resistance to apoptosis. Cancer cells have developed effective mechanisms to resist various stress-induced apoptosis (McCubrey *et al*, 2007; Djeu and Wei, 2009). Evasion of apoptosis by cancer cells differ from normal cell, which are relatively more sensitive to mitochondria-mediated apoptosis (Müllauer *et al*, 2000; Wang *et al*, 2003; Jetzt *et al*, 2009). Bcl-2 family of proteins have a key role in regulating apoptosis, where the ratio of anti-apoptotic (Bcl-2, Bcl-XL and Mcl-1) and pro-apoptotic (Bax, Bad and PUMA) members maintain integrity of the mitochondrial membrane (Kutuk and Letai, 2008). Common cancers such as breast and prostate express high levels of Bcl-XL and Bcl-2 proteins, which inhibit mitochondria-mediated apoptosis (Nuñez *et al*, 1991; Callagy *et al*, 2008). In addition to increased transcription, additional mechanisms that increase stability of anti-apoptotic proteins in cancer cells remain poorly understood (Abdelmohsen *et al*, 2007; Shimazu *et al*, 2007). Some of the known mechanisms by which proteins are stabilised in breast cancer cells are through acetylation, glycosylation or increased association with various chaperones. In MCF-7 cell line, Raf-1 exists in a complex with heat shock protein hsp-90 and this complex is essential for Raf-1 protein stability (Schulte *et al*, 1995). Another protein that gets stabilised in breast cancer is AP-2*α*, a transcription factor that binds to HER-2/neu gene promoter and has a half-life of 30 h compared with 1 h in normal cells (Li *et al*, 2006). This increased stability of AP-2*α* protein has been found to induce HER-2 overexpression, which has an adverse prognosis in 10–34% of breast cancers (Pellikainen and Kosma, 2007).

The ubiquitin–proteosome machinery, a common pathway for protein degradation, has been implicated in regulating protein stability, cell viability and apoptosis (McBride *et al*, 2003; Thompson *et al*, 2008). Proteins targeted for proteosomal degradation get polyubiquitinated at specific lysine residues through a multistep process mediated by E1, E2 and E3 ligases. The ubiquitination mechanism consists of three steps: (1) activation of ubiquitin (ub) monomers by E1 ligase, (2) transfer of activated ub from E1 ligase to E2 ligase and (3) transfer of the ub polymers from E2 ligase to the protein targeted for degradation by E3 ligase. An ubiquitin chain forming a specific lysine–lysine linkage serves as a signal for degradation of proteins (Sun, 2006; Petroski, 2008). Poorly understood aberrations in the ubiquitin–proteosome machinery could increase stability of anti-apoptotic proteins in breast cancer cells. We used cycloheximide (CHX) to inhibit *de novo* protein synthesis and investigated mechanisms that increase the stability of anti-apoptotic proteins in breast cancer cells. We find that CHX treatment promotes mitochondria-mediated apoptosis through decline in levels of Mcl-1 and Bcl-2 in normal mammary epithelial cells. In breast cancer cells, reduced ubiquitination and degradation of Mcl-1 and Bcl-2 was detected with CHX treatment. We demonstrate that unstable binding of Mcl-1 with E3 ligase Mule could be one of the prominent mechanisms that increases stability of Mcl-1 in breast cancer cells.

## Materials and methods

### Cell culture

Human breast cancer cell lines MDA-MB-231, MDA-MB-468 and MCF-7 were obtained from American Type Culture Collection (Manassas, VA, USA). These cells were cultured in DMEM containing 10 mM non-essential amino acids, 2 mM L-glutamine, 10 g ml^–1^ insulin and 10% fetal bovine serum. MCF-10A, a spontaneously immortalised untransformed human mammary epithelial cell line was obtained from Robert J Pauley (Barbara Ann Karmanos Cancer Institute, Detroit, MI, USA). HMLE, a human mammary epithelial cell line immortalised by the introduction of SV40 large-T oncogene and hTERT was obtained from Professor Robert Weinberg (Whitehead Institute at MIT, Cambridge, MA, USA). MCF-10A and HMLE were cultured in DMEM: Ham's F-12 (1 : 1) supplemented with 5% equine serum, 10 mM HEPES, 10 *μ*g ml^–1^ insulin, 20 ng ml^–1^ epidermal growth factor, 100 ng ml^–1^ cholera enterotoxin and 0.5 *μ*g ml^–1^ hydrocortisone (Pervin *et al*, 2003).

### Chemicals

Antibodies used were from the following suppliers: rabbit polyclonal anti-Ub (G0805), anti-Mcl-1 from Santa Cruz Biotech Inc. (Santa Cruz, CA, USA); rabbit polyclonal anti-Mule (A300-486A; Bethyl Laboratories, Montgomery, TX, USA), mouse anti-HRF from BD Biosciences Pharmingen (San Diego, CA, USA); rabbit anti-Puma from Abcam (Boston, MA, USA); rabbit polyclonal anti-caspase-3 (65906E), mouse monoclonal anti-cytochrome *c* (65981A) and rabbit polyclonal ERK1/2 MAP kinase (9102) from New England Biolabs (Ipswich, MA, USA); mouse monoclonal anti-Bcl-2 (Ab-1) from Calbiochem (La Jolla, CA, USA). Cycloheximide and MG132 were obtained from Sigma (St Louis, MO, USA).

### TUNEL assay

The TUNEL assay was performed using ApoAlert DNA Fragmentation Assay Kit from Clontech (Palo Alto, CA, USA). Cells (3 × 10^6^) were collected by centrifugation and washed twice with PBS. Cells were fixed in 1% formaldehyde-PBS at 4°C for 20 min and incubated with nucleotide mixture and terminal deoxynucleotidyl transferase enzyme for analysis in a Becton Dickinson Flow Cytometer (Singh *et al*, 2000).

### Caspase assay

Cells were lysed in insect cell lysis buffer containing 50 mM HEPES, 100 mM NaCl, 2 mM EDTA, 0.1% 3-((3-cholamidopropyl) dimethylammonio)-1-propanesulfonic acid (CHAPS), 10% sucrose, 5 mM DTT and 1 × protease inhibitor for 30 min at 4°C. The lysates were used for caspase-3 (30 *μ*g) and caspase-9 (50 *μ*g) enzymatic assays using respective substrates. The cleaved fluorescent substrate was quantified using a Versa Fluro flurometer (Bio-Rad, Hercules, CA, USA), with excitation at 380 nm and emission at 440 nm (Pervin *et al*, 2007).

### Cell fractionation and cytochrome *c* detection

Cytochrome *c* release into the cytosol was detected as described previously with minor modifications (Pervin *et al*, 2003; Pervin *et al*, 2007). Briefly, 6 × 10^6^ cells were harvested and washed with PBS. The cells were suspended in buffer A (20 mM HEPES-KOH (pH 7.5), 10 mM KCl, 1.5 mM MgCl_2_, 1 mM EDTA, 1 mM EGTA, 1 mM DTT, 250 mM sucrose and 1 × protease inhibitor cocktail) and were homogenised by Dounce homogeniser; unbroken cells and nuclei were removed by centrifugation at 1000 **g** for 20 min. The cytosolic fraction was analysed by western blot with an anti-cytochrome c monoclonal antibody (7H8 2C12) or with an anti-GAPDH antibody (Pervin *et al*, 2003, 2007).

### Western analysis

Cells were lysed in cell lysis buffer (50 mM HEPES, pH 7.5; 1 mM DTT, 150 mM NaCl, 1 mM EDTA, 0.1% Tween 20, 10% glycerol, 10 mM *α*-glycerophosphate, 1 mM NaF, 0.1 mM orthovanadate, 10 *μ*g ml^–1^ leupeptin, 10 *μ*g ml^–1^ aprotinin and 0.1 mM PMSF) and incubated at 4°C for 30 min for analysis of cytosolic proteins. Lysates (100 *μ*g) were resolved electrophoretically on 4–15% gradient SDS–polyacrylamide gel and electro transferred to a polyvinylidine difluoride membrane (Bio-Rad) using a tank blot procedure (Bio-Rad Mini Protean II). The membranes were incubated with various primary antibodies (1 : 1000 dilution) and respective horseradish peroxidase-linked secondary antibody (1 : 1000 dilution) (Amersham Corp., Piscataway, NJ, USA) for 1 h. Immunoreactive bands were visualised by ECL detection system (Amersham) (Singh *et al*, 2000; Pervin *et al*, 2003, 2007).

### Immunoprecipitation

Cells were lysed in lysis buffer containing 50 mM Tris/HCl, pH 8.0, 300 mM NaCl, 10 mM MgCl_2_, 0.5% Igepal Ca-630 (Sigma), 1 mM EDTA and anti-protease cocktail (Roche, San Francisco, CA, USA). Supernatant (500 *μ*g) were pre-cleared by incubation (4 h at 4°C) once with Protein A/G-agarose (Santa Cruz Biotechnology) and once with Protein A/G-agarose pre-incubated with pre-immune serum. The pre-cleared lysate was adjusted to 150 mM NaCl and 0.25% Igepal Ca-630 and subjected to overnight immunoprecipitation with the respective antibodies. The mixtures were incubated further for 4 h at 4°C with 50 *μ*l of Protein A/G-agarose and processed for western blot analysis (Pervin *et al*, 2007).

### Small-interfering (siRNA) transfection

Breast cancer cells were propagated into six-well plates at 2 × 10^5^ cells per well, grown for 24 h in complete medium followed by transfection with ON-TARGET plus Smart pool siRNA specific to Puma, HRF and Mcl-1 (Dharmacon, Chicago, IL, USA) or scrambled siRNA-negative control at a final concentration of 20 nM using lipofectamine reagent. Mule siRNA sequence (5′-AAUUGCUAUGUCUCUGGGACA-3′) used to target human Mule was designed as described before (Chen *et al*, 2005; Parsons *et al*, 2009). Efficiency of transfection was monitored by analysing the cell lysates after 3 days of transfection by western blot analysis using respective antibodies (Pervin *et al*, 2007). siRNA transected cells were further subjected to vehicle or CHX treatment for various time points, lysed and analysed by western blot analysis or enzymatic assay for caspase-3.

### Statistical analysis

Data are presented as mean±s.e.m. Student's *t*-test was performed for comparison between two groups. For statistical analysis of more than two groups, data were analysed by ANOVA, and pairwise comparisons were performed by Tukey's posttest procedure. *P*-values <0.05 were considered statistically significant.

## Results

### Resistance of breast cancer cells to CHX-induced apoptosis

**Figure 1:**
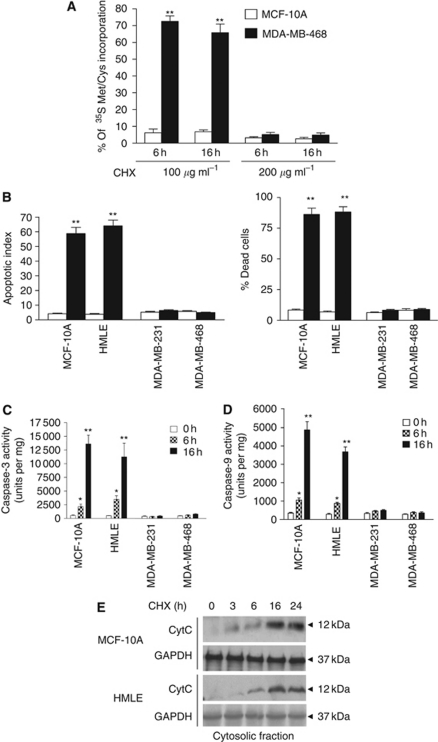
Resistance of breast cancer cells to cycloheximide (CHX) treatment. (**A**) MCF-10A and MDA-MB-468 cells treated with 100–200 *μ*g ml^–1^ of CHX for 6 or 16 h were harvested following 4 h of incubation with S^35^ Met/Cys. The cells were lysed and S^35^ Met/Cys incorporation into proteins was examined. (**B**) (Left panel) Cells treated with CHX (200 *μ*g ml^–1^) for 16 h and DNA fragmentation was assessed by TUNEL assay. (Right panel) Cells treated with CHX (200 *μ*g ml^–1^) for 16 h and cell viability was assessed by trypan blue assay, white open bar (0h); black closed bar (16h). (**C**) Cells treated with CHX (200 *μ*g ml^–1^) for 6 and 16 h were lysed and 30 *μ*g of the lysate was subjected to fluorometric assay for caspase-3 activity. (**D**) Cells treated with CHX (200 *μ*g ml^–1^) for 6 and 16 h were lysed and 50 *μ*g lysates were subjected to fluorometric assay for caspase-9 activity. (**E**) MCF-10A and HMLE cells were treated with CHX (200 *μ*g ml^–1^) for various time points (0–24 h) and subjected to mitochondria and cytosolic fractionation. Western blot analysis was performed using 100 *μ*g of cytosolic fraction using cytochrome c antibody. Data represent mean±s.e.m. of four independent experiments in triplicates (asterisks denote statistically significant values compared with untreated control group, ^*^P⩽0.05 and ^**^P⩽0.01).

We initially performed S^35^ methionine uptake assays to determine the concentrations and duration of CHX treatment required to inhibit protein synthesis in both mammary epithelial and breast cancer cells. Significant inhibition of protein synthesis was observed within 6 h of CHX treatment in the normal mammary epithelial cells (100 *μ*g ml^–1^ CHX) and breast cancer cells (200 *μ*g ml^–1^ CHX) (Figure 1A). Only in mammary epithelial cells, CHX treatment induced DNA fragmentation and cell death as detected by TUNEL assay and trypan blue exclusion method, respectively (Figure 1B). We next examined caspase-3, an executioner caspase in the apoptotic cascade that commits cells to apoptosis by inducing DNA fragmentation along with other prominent changes. Caspase-3 activity, measured using DEVD-AMC as a substrate, significantly increased in CHX-treated (6–16 h) MCF-10A and HMLE cells, while no activity was detected in breast cancer cells (Figure 1C). We also examined the activation of caspase-9, which functions upstream of caspase-3, by using Ac-LEHD-AMC as substrate. Caspase-9 activities followed the similar pattern as caspase-3 and increased 5–6 folds at 16 h only in CHX-treated MCF-10A and HMLE cells, while no significant increased activity was detected in breast cancer cells (Figure 1D). Breast cancer cell lines became cytostatic with CHX treatment at 24 h and no significant increase in caspase-3/9 activities or apoptosis was detected at 16 h (Figures 1B–D) or even 24–48 h (data not shown). Cytostatic cells were viable even until 72 h of CHX treatment as assessed by trypan blue exclusion assay and no TUNEL-positive cells were detected in that time frame (data not shown).

To determine whether CHX-induced increase in caspase-3 and caspase-9 activities were mitochondria dependent in normal mammary epithelial cells, the cells were fractionated and the cytosolic fractions were analysed for an increase in cytochrome c levels by western blot analysis. We found increased cytochrome c levels at 16 h in the cytoplasm of CHX-treated MCF-10A and HMLE cells, confirming the involvement of mitochondria-mediated apoptotic pathway (Figure 1E). No increase in cytosolic cytochrome *c* was found in breast cancer cells even at 48 h of CHX treatment (data not shown). Our data therefore indicate that *de novo* inhibition of protein synthesis induces mitochondria-mediated apoptosis only in normal mammary epithelial cells, while breast cancer cells were resistant to this treatment.

### Reduced ubiquitination and increased stability of anti-apoptotic proteins in cancer cells

**Figure 2:**
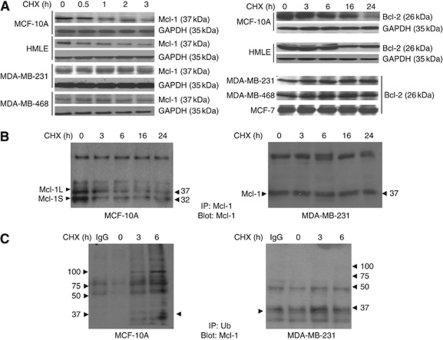
Reduced ubiquitination and increased stability of Mcl-1 and Bcl-2 in breast cancer cells treated with CHX (200 *μ*g ml^–1^). (**A**) Cells treated with CHX for various time points and cell lysates (100 *μ*g) were subjected to western blot analysis for Mcl-1 and GAPDH (left panel) and Bcl-2 and GAPDH (right panel) proteins. (**B**) CHX-treated cell lysates (500 *μ*g) from MCF-10A (left panel) and MDA-MB-231 (right panel) were subjected to immunoprecipitation with anti-Mcl-1 antibody and immunoblotted for Mcl-1. (**C**) CHX-treated cell lysates (500 *μ*g) from MCF-10A (left panel) and MDA-MB-231 (right panel) were subjected to immunoprecipitation with anti-Ub antibody and immunoblotted for Mcl-1.

Since CHX treatment increased cytosolic cytochrome c in MCF-10A and HMLE cells, we examined the stability of Mcl-1 and Bcl-2 proteins that maintain the integrity of mitochondrial membrane. The stability of anti-apoptotic proteins Mcl-1 and Bcl-2 was also assessed in these MDA-MB-468, MDA-MB-231 and MCF-7 breast cancer cells after treatment with CHX (200 *μ*g ml^–1^). Mcl-1 levels were reduced in CHX-treated MCF-10A (52±6%) and HMLE (62±6%) cells at 3 h (Figure 2A, left panel); whereas, Bcl-2 levels were reduced by 66±7% in MCF-10A and 48±6% in HMLE cells after similar treatments at 16 h as assessed by western blot analysis (Figure 2A, right panel). No significant decline in the levels of Mcl-1 and Bcl-2 was observed in the cancer cell lines where the overall levels remained relatively stable and even increased at 16–24 h of CHX treatment (Figure 2A, left and right panels). The decline in Mcl-1 and Bcl-2 in CHX-treated normal cells was further confirmed by immunoprecipitation assays. Mcl-1 and Bcl-2 were immunoprecipitated with Mcl-1 or Bcl-2 antibodies from CHX-treated lysates, which were further immunoblotted with their respective antibodies. Immunoprecipitation analysis confirmed that the decline in Mcl-1 (Figure 2B, left panel) and Bcl-2 (data not shown) was only in CHX-treated normal mammary epithelial cells but not in the CHX-treated breast cancer cells (Figure 2B, right panel).

We next examined the mechanism(s) that promote selective decline of Mcl-1 and Bcl-2 only in MCF-10A and HMLE cells. To determine whether the ubiquitination of Mcl-1 and Bcl-2 increased with CHX treatment, the cell lysates at various time points were immunoprecipitated with ubiquitin antibody and immunoblotted for Mcl-1. We found increased levels of ubiquitinated Mcl-1 in CHX-treated MCF-10A at 16–24 h (Figure 2C, left panel), while in MDA-MB-468 cells, no prominent ubiquitination of Mcl-1 was detected (Figure 2C, right panel). Similar results were obtained when total cellular ubiquitinated proteins were immunoprecipitated and immunoblotted with Bcl-2. Ubiquitinated Bcl-2 was found only in CHX-treated MCF-10A cells but not in MDA-MB-231 cells (data not shown).

**Figure 3:**
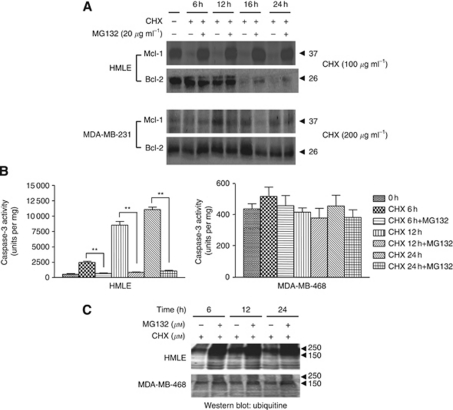
Proteosome-mediated degradation of Mcl-1 and Bcl-2 in HMLE cells can be attenuated by proteosome inhibitor MG132. (**A**) CHX-treated cells were treated with or without MG132 and subjected to western blot analysis using anti-Mcl-1 and anti-Bcl-2 antibodies. (**B**) CHX-treated cells were treated with or without MG132 and subjected to fluorometric assay for caspase-3 activity. Data represent mean±s.e.m. of three independent experiments done in quadruplicates (the asterisks denote statistical significant values compared with CHX+MG132 treatment groups, ^**^P⩽0.01. (**C**) CHX-treated cells were treated with or without MG132 and subjected to western blot analysis using anti-Ub antibody.

To further determine whether ubiquitinated Mcl-1 and Bcl-2 in normal mammary epithelial cells were degraded through the 26S proteosomal complex, a chemical inhibitor of the proteosomal machinery, MG132 was used. The stability of Bcl-2 and Mcl-1 proteins was examined by western blotting in cells pre-incubated with MG132 for 2 h followed by CHX treatment. Treatment with MG132 inhibitor completely attenuated the decline of Mcl-1 in CHX-treated human mammary epithelial cells, confirming the involvement of the 26S proteasomal degradation pathway (Figure 3A). However, CHX-induced decline of Bcl-2 levels was only partially attenuated with MG132 treatment in the normal mammary epithelial cells (Figure 3A). Our data, therefore, may suggest that MG132 selectively inhibits CHX-induced degradation of Mcl-1 but Bcl-2 degradation in response to CHX may not be entirely dependent on proteasomal pathways in mammary epithelial cells. Pre-treatment with the inhibitor MG132 followed by CHX exposure resulted in no significant change in the Mcl-1 or Bcl-2 levels in the breast cancer cell lines MDA-MB-231 (Figure 3A), MDA-MB-468 and MCF-7 (data not shown). Pre-treatment with the inhibitor, MG132, also attenuated the CHX-induced dramatic increase in caspase-3 activity in MCF-10A cells (Figure 3B, left panel). On the other hand, in breast cancer cells, no significant change in caspase-3 activity was observed with or without MG132 pre-treatment followed by CHX exposure (Figure 3B, right panel). Furthermore, we detected higher levels of polyubiquitinated proteins that accumulate without degradation in MG132-treated MCF-10A but not in MDA-MB-468 cells (Figure 3C). Therefore, our results indicate that inhibition of protein synthesis in MCF-10A and HMLE cells increases rapid degradation of Mcl-1 and to some extent Bcl-2 by ubiquitin–proteosome machinery.

### Reduced association of Mcl-1 with Mule in breast cancer cells

**Figure 4:**
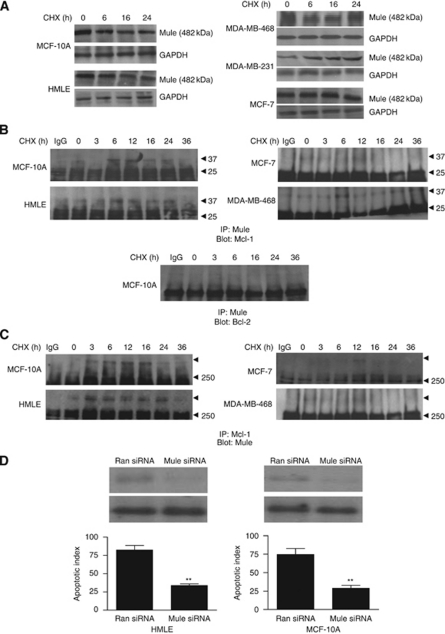
Reduced Mule/Mcl-1 complex detected in breast cancer cells compared with mammary epithelial cells. (**A**) Mammary epithelial cells (MCF-10A and HMLE, left panel) and breast cancer cells (MDA-MB-468, MDA-MB-231 and MCF-7) were treated with CHX (200 *μ*g ml^–1^) for various time points (0–24 h) and 100 *μ*g of protein lysates were immunoblotted using anti-Mule antibody. (**B**) (Top panel) Mammary epithelial cells (MCF-10A and HMLE, left panel) and breast cancer cells (MCF-7 and MDA-MB-468, right panel) were treated with CHX (200 *μ*g ml^–1^) for various time points (0–36 h) and immunoprecipitated with anti-Mule antibody or with IgG (16 h) and immunoblotted for Mcl-1. (Bottom panel) MCF-10A cells were treated with CHX (200 *μ*g ml^–1^) for 0–36 h and immunoprecipitated with anti-Mule antibody or with IgG (16 h) and immunoblotted for Bcl-2. (**C**) Mammary epithelial cells (MCF-10A and HMLE, left panel) and breast cancer cells (MCF-7 and MDA-MB-468) were treated with CHX (200 *μ*g ml^–1^) for various time points (0–36 h) and immunoprecipitated with anti-Mcl-1 antibody or with IgG (16 h) and immunoblotted for Mule. (**D**) (Top panel) Small-inhibitory RNA (siRNA)-mediated inhibition of Mule in mammary epithelial cells (HMLE and MCF-10A). Western blot analysis showing Mule (top) and GAPDH (bottom). (Bottom panel) Measurement of apoptotic index in random (Ran) and Mule siRNA (Mule siRNA) treated mammary epithelial cells by TUNEL assay after CHX treatment. ^**^P<0.01.

We next examined the key players or mechanisms that increased the ubiquitination and degradation of Mcl-1 and Bcl-2 in mammary epithelial cells as well as their aberrations in breast cancer cells. E3 ligase, which targets proteins for degradation, has been characterised for Mcl-1 but not for Bcl-2. We examined the expression of Mule, the specific E3 ligase for Mcl-1 by western blot analysis in both the normal mammary epithelial and breast cancer cells. Mule was constitutively expressed in normal mammary epithelial and breast cancer cells (in some breast cancer cell lines Mule was induced by CHX treatment). The protein level of Mule was also comparable between MCF-10A and MDA-MB-468 cells, while it was induced in MDA-MB-231 cells at 6–16 h of CHX treatment (Figure 4A). The mRNA expression of Mule as determined by real-time qPCR in all the cell lines were examined, which followed similar pattern as that of the proteins (data not shown).

Since ubiquitination of Mcl-1 was reduced in breast cancer cells even though Mule was constitutively expressed or induced by CHX, we examined the association of Mcl-1 and Mule by immunoprecipitation assays. Mule was immunoprecipitated from CHX-treated cell lysates of MCF-10A and MCF-7 cells and immunoblotted with Mcl-1 antibody. In MCF-10A and HMLE cells, we found increased Mcl-1 and Mule complex between 6–24 h of CHX treatment (Figure 4B, left panel). We also performed immunoprecipitation of CHX-treated MCF-10A and MCF-7 cells by Mule followed by western blotting with anti-Bcl-2 antibody. We did not find any significant association of Mule with Bcl-2 in MCF-10A cells (Figure 4B, bottom panel) or in MCF-7 cells (data not shown). We also confirmed this prolonged association between Mule and Mcl-1 in CHX-treated normal mammary epithelial cells by immunoprecipitating Mcl-1 and blotting for Mule (Figure 4C, left panel). However, in all the breast cancer cell lines examined, we were unable to detect any prolonged association of Mcl-1 and Mule between 0 and 36 h of CHX treatment. In MDA-MB-468 cells, we could detect only transient association of Mcl-1 and Mule at 12–16 h, and at each of these time points where the complex was detected, there was a sharp decline in Mule–Mcl-1 complex (Figure 4B, right panel). In MCF-7 cells, we could detect association between Mcl-1 and Mule only at 12 h (Figure 4C, right panel); while in MDA-MB-231 cells, very weak association was detected at 24 and 48 h (data not shown). Our results indicate that unstable association between Mule and Mcl-1 may contribute to decreased ubiquitination and degradation of Mcl-1 in breast cancer cells. We were unable to detect significant association of another known E3 ligase *β*-TrCP with Mcl-1 in either normal mammary epithelial or breast cancer cells (data not shown).

In order to further investigate the role of Mule during CHX-induced apoptosis in normal mammary epithelial cells, we downregulated Mule expression by siRNA. HMLE and MCF-10A cells treated with either random siRNA or Mule siRNA were incubated with CHX (200 *μ*g ml^–1^) and apoptotic index was analysed by TUNEL assay. We found that inhibition of Mule levels in these cells by siRNA treatment significantly decreased their apoptotic index (HMLE: 61±5% MCF-10A: 66±3%) (Figure 4D).

### CHX regulation of Mcl-1-binding proteins Puma and HRF

**Figure 5:**
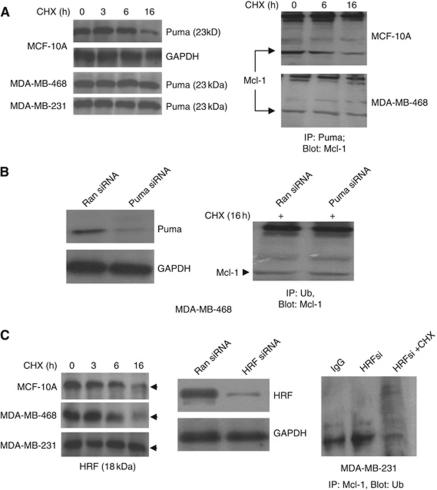
Downregulation of HRF increased Mcl-1 ubiquitination. (**A**) (Left panel) Cells treated with CHX (200 *μ*g ml^–1^) for various time points (0–16 h) and 100 *μ*g of cell lysates were subjected to immunoblot analysis using anti-Puma antibody. (Right panel) Cells treated with CHX for various time points (0–16 h) were immunoprecipitated for Puma and immunoblotted with anti- Mcl-1 antibody. (**B**) MDA-MB-468 cells treated with scrambled (Ran siRNA) or HRF siRNA was immunoblotted for Puma and GAPDH (left panel), or immunoprecipitated with ubiquitin (Ub) antibody and blotted for Mcl-1 (right panel). (**C**) (Left panel) Cells treated with CHX (200 *μ*g ml^–1^) for various time points (0–16 h) and 100 *μ*g of cell lysates were subjected to immunoblot analysis using anti-HRF antibody. (Middle panel) MDA-MB-231 cells treated with scrambled (Ran siRNA) or HRF siRNA and immunoblotted using anti-HRF antibody. (Right panel) Cells treated with HRF siRNA (HR si) or with HRFsi+CHX (200 *μ*g ml^–1^) were immunoprecipitated with Mcl-1 antibody and immunoblotted using anti-Ub antibody.

Puma and HRF (translationally controlled tumour protein) are known to interact with Mcl-1 and increase the stability of Mcl-1. Accordingly, we initially examined the stability of Mcl-1 associating proteins in CHX-treated mammary epithelial and breast cancer cells. Puma (Figure 5A) and HRF (Figure 5C) levels declined with CHX treatment in MCF-10A cells. In most of the CHX-treated breast cancer cell lines, we found increased stability of Mcl-1 associating proteins Puma (Figure 5A, left panel) and HRF (Figure 5C). We further examined the association of Puma, and HRF with Mcl-1 by immunoprecipitating each of these proteins and western blotting for Mcl-1. Since Puma levels declined in CHX-treated MCF-10A cells, we further examined the effects of CHX on Puma–Mcl-1 complexes by immunoprecipitating total cytosolic Puma and immunoblotting for Mcl-1. We found Puma associated with Mcl-1 in control MCF-10A cells but the complex was reduced in CHX-treated cells (Figure 5A, right panel). In both control and CHX-treated MDA-MB-468 cells, Puma–Mcl-1 complex remained stable (Figure 5A, right panel). To examine whether a decline in Puma could promote Mcl-1 ubiquitination and degradation, we downregulated Puma in breast cancer cell lines with Puma siRNA and examined Mcl-1–Mule complex. Puma levels reduced by 70% in MDA-MB-468 cells treated with Puma siRNA compared to those treated with scrambled controls (Figure 5B, left panel). A decline in Puma levels did not increase Mcl-1–Mule complex (data not shown) or ubiquitination of Mcl-1 as assessed by immunoprecipitation and western analysis in breast cancer cells (Figure 5B, right panel). While the levels of HRF declined with CHX treatment in MCF-10A, its level did not change in MDA-MB-231 cells. However, MDA-MB-468 cells behaved similar to normal mammary cells in response to CHX (Figure 5C, left panel). We also downregulated Mcl-1-associated HRF in breast cancer cell lines MDA-MB-231 with HRF siRNA (Figure 5C, middle panel) and found a moderate increase in ubiquitination of Mcl-1 levels (Figure 5C, right panel).

### Downregulation of pERK1/2 increased Mcl-1–Mule complex in breast cancer cells

**Figure 6:**
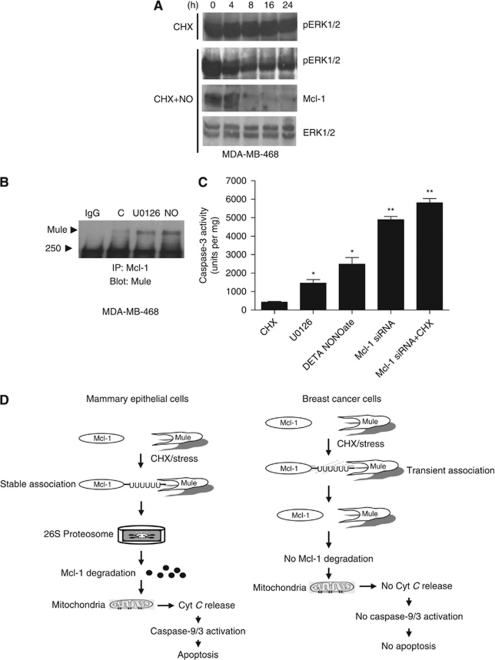
Downregulation of ERK1/2 increases apoptosis in breast cancer cells. (**A**) MDA-MB-468 cells were treated with either CHX, alone or in combination with DETA-NONOate, a nitric oxide (NO) donor for 24 h and immunoblotted using anti-pERK1/2, ERK1/2 or Mcl-1 antibodies. (**B**) Cells treated with U0 126 (10 *μ*g ml^–1^), DETA-NONOate, 1 mM) or IgG for 48 h were lysed, immunoprecipitated with Mcl-1 antibody and blotted for Mule. (**C**) MDA-MB-468 cells were subjected to various treatments and 30 *μ*g of total protein lysates were analysed for caspase-3 enzymatic activity. (**D**) Flow diagram explaining sensitivity of mammary epithelial cells or resistance of cancer cells to CHX treatment.

It has been shown that ERK1/2 can phosphorylate and stabilise Mcl-1 in breast cancer cells (Ding *et al*, 2008). We found ERK1/2 and its active form pERK1/2 upregulated in all the breast cancer cells examined and their levels remain unchanged with CHX treatment, indicating increased stability of these proteins (Figure 6A). Inactivation of pERK1/2 with MEK inhibitor U0 126 (data not shown) or nitric oxide donor DETA-NONOate promoted the decline of Mcl-1 levels in breast cancer cell line MDA-MB-468 (Figure 6A). Downregulation of pERK1/2 by treatment with MEK inhibitor or nitric oxide donor also increased Mule–Mcl-1 (Figure 6B, left panel) complex and caspase-3 activity in breast cancer cells, indicating induction of apoptosis (Figure 6C, right panel). However, MEK1/2 inhibitor or nitric oxide induced delay and lower caspase-3 activity in cells compared with higher caspase-3 activation when Mcl-1 was downregulated by Mcl-1 siRNA (Figure 6C, right panel). These results indicate that HRF and ERK1/2 partially increased the stability of Mcl-1 in breast cancer cells. It is possible that additional mechanisms operate in breast cancer cells that increase stability of Mcl-1 and resistance to apoptosis.

## Discussion

In this study, we found that increased stability of anti-apoptotic proteins like Mcl-1 and Bcl-2 rendered breast cancer cells resistant to CHX-induced apoptosis. However, in mammary epithelial (MCF-10A, HMLE) cells, CHX treatment increased degradation of Mcl-1 through the ubiquitin-dependent proteosomal pathway to induce apoptosis. We show that downregulation of Mcl-1 by Mcl-1 siRNA or inhibiting ERK1/2 through U0126 or nitric oxide treatment increased the sensitivity of breast cancer cells to CHX-induced apoptosis. Our result is consistent with a number of studies which show that drugs like flavopiridol, lipoxygenase inhibitors and roscovitine downregulated both Mcl-1 and Bcl-2 in breast cancer cells, while okadaic acid and immunotoxins among others specifically downregulated only Mcl-1 to induce apoptosis (Rieber *et al*, 2002; Tong *et al*, 2002; Wittmann *et al*, 2003; Andersson *et al*, 2004; Ortiz-Ferrón *et al*, 2008).

We found reduced ubiquitination of Mcl-1 and unstable association of Mcl-1 with its specific E3 ligase Mule in breast cancer cells treated with CHX. To our knowledge, this is the first report that has found transient association between Mule and Mcl-1 in breast cancer cells. Mule is a novel ubiquitin that contains domain homologous to E6-AP carboxyl terminus (HECT) as well as Bcl-2 homology region (BH3) domain that allows Mule to specifically interact with Mcl-1 (Warr *et al*, 2005; Zhong *et al*, 2005). We were able to detect a stable prolonged association between Mcl-1–Mule in CHX-treated MCF-10A cells, where Mcl-1 undergoes prominent ubiquitination and degradation.

Mcl-1 is stabilised by HRF, which serves as Mcl-1 chaperone and prevents its proteasomal degradation (Liu *et al*, 2005). Mcl-1 associating protein Puma (p53 upregulated modulator of apoptosis) also increases Mcl-1 stability, but does not ultimately prevent Mcl-1 degradation since its PEST (praline, glutamic acid, serine and threonine) region is still exposed (Mei *et al*, 2005). Mcl-1 also interacts with tankyrase-1, which promotes telomere elongation in human cells, and its overexpression inhibits the activity of Mcl-1L and Mcl-1S (Bae *et al*, 2003). In our study, downregulation of Puma did not decrease Mcl-1 levels or increase CHX-mediated apoptosis in breast cancer cells. We did find downregulating HRF in breast cancer cells increased Mcl-1 ubiquitination and apoptosis; however, higher apoptosis induction was observed when Mcl-1 was directly downregulated by Mcl-1 siRNA or indirectly by downregulating pERK1/2. This suggests that mechanisms other than Mcl-1 associating proteins could be operating in breast cancer cells leading to increased stability of Mcl-1.

Although we found that reduced 26S proteosomal degradation of key anti-apoptotic proteins increases survival of breast cancer cells, highly selective potent inhibitors of this machinery is emerging as promising new anti-tumour agents (Cardoso *et al*, 2004; Sterz *et al*, 2008). The rational that promoted this therapeutic approach was that increased stabilisation of Rpn4, a transcription activator of proteasome genes, severely reduced cell viability under stressed conditions. Rpn4, in addition to being a major mediator of core Rpn4-proteosome feedback circuit also appears to be a major mediator in a stress response network (Xie, 2010). Bortezomib, a proteasome inhibitor, has been shown to have some beneficial effect of in reducing proliferation and metastatic potential of aggressive breast cancer cell lines in *in vitro* and *in vivo* model (Jones *et al*, 2010). However, this compound, which is also the only FDA-approved proteasome inhibitor in clinical use, has shown limited clinical activity against metastatic breast cancer in patients (Yang *et al*, 2006). Bortezomib, however, has proven effective against some haematologic malignancies like lymphoma where it decreases proliferation, induces apoptosis and enhances sensitivity to various chemotherapy and radiation treatments (Ludwig *et al*, 2005; Cavo, 2007). Lack of effectiveness of proteosome inhibitors against breast cancer warranted using it in combination with standard cytotoxic agents including docetaxel. A clinical trial using Bortezomib and docetaxel combination as treatment regimen against malignant breast cancer in patients has shown very limited clinical efficacy (Awada *et al*, 2008). Our current findings may suggest that increasing 26S proteosome-mediated degradation of Mcl-1 might have therapeutic benefits in inducing apoptosis in breast cancer cells (Figure 6D).

Since we found transient association between Mcl-1 and Mule in cancer cells, in addition to HRF and pERK1/2 we are examining deubiquitinating enzymes (DUBs) that function downstream in ubiquitin pathways and have the potential to be the final editors of protein ubiquitination status and thus determine substrate fate (Millard and Wood, 2006). In several studies, DUBs have been implicated in regulating the stability of several proteins and can extend the half-life of proteins destined for proteasomal degradation by catalysing hydrolysis of isopeptide bond in ubiquitin protein conjugates (Graner *et al*, 2004; Sheng *et al*, 2006; Pereg *et al*, 2010). Some of these DUBs are overexpressed in breast cancer (McFarlane *et al*, 2010).

Few studies have correlated increased stability of proteins with oncogenicity or tumour progression. Signalling cascades in cancer cells that increase resistance to apoptosis has been targeted to control tumourigenesis. Our finding of increased stability of Mcl-1 in breast cancer cells due to reduction of Mcl-1–Mule complex introduces new players that can be targeted for therapeutic interventions to induce apoptosis in breast cancer. Further studies focusing on better understanding of deubiquitination–ubiquitin cycle's role are needed to develop new therapeutic targets for breast cancer therapy.
